# Untangling the Reaction Mechanism of the Polysaccharide Lyase PL42 Using QM/MM Metadynamics Simulations

**DOI:** 10.1021/acs.jcim.6c00945

**Published:** 2026-04-17

**Authors:** Santiago Alonso-Gil, Tatsuya Kondo, Shinya Fushinobu, Pedro A. Sánchez-Murcia

**Affiliations:** 1 Laboratory of Computer-Aided Molecular Design, Division of Medicinal Chemistry, Otto-Loewi Research Center, Medical University of Graz, Neue Stiftintalstr. 6/III, Graz A-8010, Austria; 2 56053 Institut Laue-Langevin, Grenoble 38042, France; 3 Department of Applied Biological Chemistry, Graduate School of Agriculture, Osaka Metropolitan University, Sakai, Osaka 599-8531, Japan; 4 Department of Biotechnology, 13143 The University of Tokyo, Bunkyo-ku, Tokyo 113-8657, Japan; 5 Collaborative Research Institute for Innovative Microbiology, 13143 The University of Tokyo, Bunkyo-ku, Tokyo 113-8657, Japan; 6 BioTechMed-Graz, Mozartgasse 12/II, Graz A-8010, Austria

## Abstract

Polysaccharide lyase family 42 (PL42) enzymes cleave terminal Rha-GlcA motifs from gum arabic through β-elimination. However, the neutron-based catalytic proposal for FoRham1 has not been validated on a free-energy surface. Here, we combine QM/MM well-tempered metadynamics with umbrella sampling to analyze the reaction mechanism of the PL42 lyase FoRham1 at the atomic level. The calculations support a concerted yet highly asynchronous syn β-elimination, where His85 functions as the catalytic base/acid, with assistance from Asp83 and doubly protonated His105. Arg166 stabilizes the glucuronate carboxylate electrostatically. The reactive substrate conformation is an inverse-chair ^1^ *C*_4_ state, and the +1 sugar follows a ^1^ *C*_4_ → ^1^ *C*_4_ → ^1^ *H*_2_ pathway along the minimum-free-energy route. The metadynamics barrier (∼19 kcal mol^–1^) is refined to 17.7 kcal mol^–1^ through umbrella sampling, aligning reasonably with experimental data. Beyond confirming the proposed experimental mechanism, the results demonstrate that PL42 catalyzes β-elimination via a histidine-based catalytic system distinct from those of PL7 and PL38. Additionally, they expand the emerging conformational framework linking polysaccharide lyase mechanisms through their product-ring geometries.

## Graphical abstract

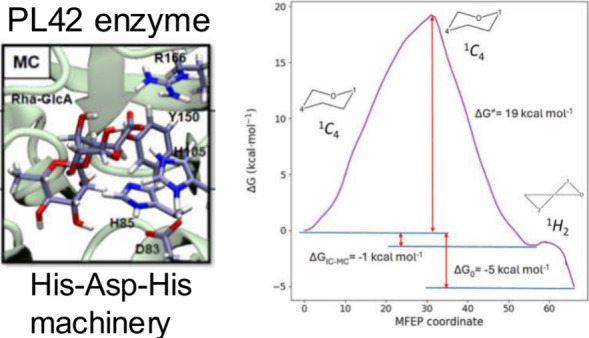

## Introduction

Polysaccharide lyases (PLs) play a crucial role in the degradation and modification of complex carbohydrates, including glycosaminoglycans and plant cell wall polysaccharides. PLs catalyze the cleavage of uronic acid-containing polysaccharides via a β-elimination mechanism, generating unsaturated oligosaccharide products that are valuable for biochemical and structural studies.^1^ Among the polysaccharide lyase families, the recently defined PL42 family has attracted interest for its distinctive substrate specificity and catalytic mechanism. This family includes L-rhamnosyl-α-1,4-d-glucuronate (Rha-GlcA) lyases, which specifically target the rhamnosyl-glucuronate linkage, an important structural component of certain polysaccharides.^2^

More broadly, polysaccharide lyases are relevant because they enable controlled, selective depolymerization of uronic acid-containing polysaccharides under mild conditions, which in turn supports both fine-structure analysis and the rational production of bioactive oligosaccharides. Recent reviews on polysaccharide degradation emphasize that partial enzymatic depolymerization is indispensable for connecting backbone architecture and molecular conformation with downstream properties and bioactivities.^3^ In the alginate field, this mechanistic value is coupled to an important preparative role: recent studies have characterized lyases that enrich trisaccharide products or selectively target polyM segments, illustrating how substrate specificity and action mode can be leveraged to obtain better-defined oligosaccharide mixtures.^4,5^

These features explain the current scientific interest in PLs across food, agricultural, marine, and biomedical research. Alginate oligosaccharides obtained through lyase-based processes are being explored for antioxidant, immunomodulatory, antimicrobial, antitumor, antihypertensive, antidiabetic, prebiotic, and plant-growth-related activities, while the continued discovery of new enzyme families expands the toolbox available for such applications.^4,6,8^ The field is also moving toward translational settings: for example, a polysaccharide lyase from Stenotrophomonas maltophilia was recently shown to inhibit and degrade acetylated alginate from clinical *Pseudomonas aeruginosa* isolates, underscoring the potential of PLs as antibiofilm adjuvants in infection control.^7^

Within this broader landscape, PL42 remains comparatively underrepresented in the literature. To date, discussion of the family is centered mainly on the work that established gum arabic L-rhamnose-α-1,4-d-glucuronate lyases as a distinct PL family and the subsequent neutron crystallographic study that resolved the active-site protonation states and catalytic architecture of FoRham1.^2,9^ This limited but high-quality body of work makes PL42 especially interesting from a mechanistic standpoint, because it highlights a chemically distinct substrate class and suggests a catalytic solution that is not captured by the better-studied PL7 and PL38 systems.

Gum arabic (GA), a natural exudate from Acacia trees, is a highly branched polysaccharide composed primarily of galactose, arabinose, rhamnose, and glucuronic acid residues.^10^ GA has various industrial applications, including its use as an emulsifier and stabilizer in the food and pharmaceutical industries. The nonreducing ends of GA side chains are frequently capped with Rha-GlcA moieties, making it a prime substrate for PL42 lyases.^2^ The enzymatic cleavage of these linkages not only facilitates the structural characterization of GA but also holds potential for modifying its physicochemical properties for industrial applications.

Recent X-ray and neutron diffraction studies have yielded high-resolution structural insights into the catalytic mechanism of PL42 lyases.^2,9^ These structures show an active site organized around a conserved His–His–Asp triad that supports charge neutralization and proton transfer during the β-elimination step. In particular, the neutron crystallographic analysis by Yano et al.^9^ proposed a detailed mechanism for the GA-specific L-rhamnose-α-1,4-d-glucuronate lyase from *Fusarium oxysporum* (FoRham1). In this model, His85 functions as the general base/acid: it abstracts the C5 proton of the glucuronate, triggering electron redistribution that promotes syn β-elimination (Figure 1). Additional residues help stabilize the reactive configuration: Arg166 provides electrostatic stabilization of the uronate carboxylate, His105 supports proton transfer, and Tyr150 also interacts with the substrate carboxylate. As observed in other uronate-active enzymes,^11^ the definition of a distinctive hydrogen-bonding network supports precise substrate positioning and transition-state stabilization.

**Figure 1:**
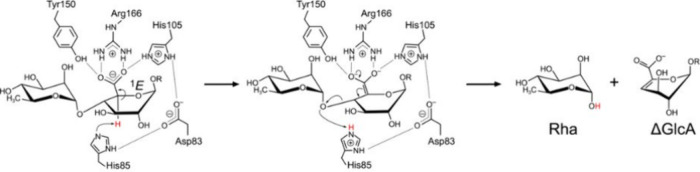
Proposed syn β-elimination mechanism for the FoRham1 enzyme (PL42) and proposed reaction mechanism for FoRham (PL7). Figure adapted from references 2 and 9 with permission of the authors.

Previous quantum mechanics/molecular mechanics (QM/MM) simulations for the family of PL7 alginate lyases pointed to a concerted, yet asynchronous, syn β-elimination in which the Tyr residue (Tyr150 in FoRham1) serves as base/acid, and the +1 sugar undergoes skew-boat distortion, with a carbanion-like transition state.^12^ In contrast, integrated crystallographic and QM/MM analyses of the Bacteroides ovatus PL38 lyase (BoPL38) indicate that Y298 and H243 abstract the H5 proton, promoting syn- and anti-β-elimination, respectively, from a preactivated/distorted +1 sugar and through distinct transition states.^13^ Here, we apply these principles to PL42, which features a distinct His–His–Asp catalytic apparatus.

Importantly, this study represents the first application of QM/MM simulations to investigate the catalytic mechanism of a PL42 α-L-rhamnosyl-(1→4)-d-glucuronate lyase (EC 4.2.2.28) from *Fusarium oxysporum* (FoRham1 enzyme). Our goals are 3-fold: to test whether FoRham1 indeed follows the syn β-elimination mechanism inferred from neutron crystallography, to determine the catalytically competent conformation of the +1 glucuronate, and to place PL42 within the emerging conformational framework that relates the product-ring geometries of different PL families. QM/MM simulations, including both metadynamics- and umbrella-sampling-based approaches, are versatile tools to elucidate the reaction mechanisms of carbohydrate-active enzymes, providing a strong basis for the present work.^14–19^ Combining QM/MM modeling and enhanced sampling enables us to explore the conformational dynamics, energy landscapes, and potential transition states associated with the catalytic process, providing complementary insights to experimental structural data. This underscores the novelty of the present work and advances the atomic-level understanding of polysaccharide lyase mechanisms.

## Methods

### Model Construction

By working with the coordinates of the joint XN structure of FoRham1 obtained by Yano et al. (PDB ID 7YQS),^9^ we reconstructed a FoRham1 + Rha-GlcA complex. Rha-GlcA was placed manually rather than docked because the available X-ray and neutron structures already define the binding pose at subsites −1/+1 more tightly than a generic docking protocol would for this highly charged carbohydrate. We therefore preserved the crystallographic rhamnose pose, overlapped the carboxylate group of the +1 sugar with the crystallographic acetate/carboxylate small substrate, retained the experimentally observed catalytic contacts, and then fully relaxed the resulting complex by extensive classical MD before starting the QM/MM stage. The original structure with 419 amino acids, one Ca2+ and one Na+, and 297 water molecules (from the 299 original ones, two water molecules were removed to allocate the d-glucuronate moiety) in complex with the substrate (original L-rhamnose moiety in subsite −1, original acetate group forming part of a ^4^ *C*_1_ conformer of the d-glucuronate) was adequately protonated at the physiological pH of the crystallization. His53, His85, His104, His136, and His299 were δ-protonated, His99, His178, His229, His288, His303, and His390 were ε-protonated, and His105 was doubly protonated (positively charged) as experimentally observed. Because neutron crystallography directly informed the active-site protonation pattern, especially for His105, we did not reassign the catalytic residues using empirical p*K*_a_ predictors. All Asp and Glu residues were kept negatively charged, and all Lys and Arg residues were kept positively charged. The system was solvated in an octahedral box of water molecules, and the total net charge (−11 e) was neutralized with an extra 11 Na+ cations. In total, our model contained 419 protein residues, 1 Rha-GlcA molecule, 1 Ca2+, 12 Na+, and 14587 water molecules forming an 18.774 nm × 18.774 nm × 18.774 nm octahedral box (Figure S1).

### Classical Molecular Dynamics Simulations

All classical molecular dynamics (cMD) simulations were performed with AMBER 20.^20^ A FoRham1 + Rha-GlcA model was parametrized using the ff19SB force field^21^ for protein residues and solvated ions. The Rha-GlcA parametrization was obtained by optimizing the undistorted disaccharide, calculating the restrained electrostatic potential (RESP) charges, and following the standard procedure using the antechamber software. The water molecules were described as TIP3P molecules.^22^ We retained TIP3P to preserve direct compatibility with the established AMBER/PLUMED/TeraChem workflow used throughout this study. The solvated system was minimized in three consecutive steps (all hydrogens, solvent and all system) and heated up in another three consecutive steps of 50 ps (time step 0.5 fs) from 0 to 100 K, from 100 to 200 K, and from 200 to 300 K using the Langevin thermostat (NVT ensemble, gamma friction coefficient of 1.0 ps-1). The simulation ensemble was then changed to NPT at 1 atm, and the density of the system was stabilized in a 1 ns simulation (time step 2 fs, SHAKE algorithm to restrain the C–H vibrations). Finally, a 50 ns equilibration was run until the root-mean-square deviation (RMSD) of the protein stabilized (Figure S2, RMSD ∼1.1 Å for the whole protein, no hydrogens). Only structures from this equilibrated regime were used to initiate the QM/MM stage.

### QM/MM Well-Tempered Metadynamics

All QM/MM^23^ well-tempered^24^ metadynamics^25^ simulations were performed with AMBER 20, coupled with PLUMED 2.7 ^26^ and TeraChem.^27^ The QM region (Figure S3) is formed by all the atoms of the Rha-GlcA substrate (charge −1) and the side chains (cutting the CA-CB bond) of D83 (charge −1), H85 (neutral), H105 (charge +1), and Y150 (neutral). The QM region is described using the PBE functional^28^ with the def2-SVP basis set^29^ and a D3 dispersion correction.^30^ Before starting the metadynamics, the last point of the cMD simulation was equilibrated by running 10 ps (1 fs time step) of QM/MM MD. To reconstruct the conformational free-energy landscape (FEL) of the 6-membered ring of GlcA, we defined the Cremer-Pople puckering coordinates (φ, θ) of the ring as collective variables. The shape of the Gaussian potentials was 0.1 rad × 0.1 rad × 0.5 kcal/mol, and one Gaussian was added every 100 MD steps. The well-tempered parameter was 15 kcal/mol. After adding 854 Gaussian potentials (Figure S4, 85.4 ps total simulation time), the system evolved back and forth from the ^5^ *S*_1_ to the ^1^ *C*_4_ region, passing by the °*S*_2_ as well. After this intensive exploration, we considered the free-energy surface converged.

Once we found the most stable conformation of the GlcA moiety (^1^ *C*_4_), a representative structure taken from the metadynamics trajectory was equilibrated along 10 ps of QM/MM MD, and a second well-tempered metadynamics simulation was started to reconstruct the free-energy landscape of the β-elimination mechanism hypothesized by Yano et al.^9^ The mechanism proceeds via the formation of an intermediate in which the H5 of GlcA is transferred to the basic nitrogen of H85. Once H85 is protonated, the same hydrogen is shared with the glycosidic oxygen, activating cleavage of the glycosidic bond. To simulate this process, two collective variables were defined (CV1, CV2, Figure 2). CV1 represents the proton transfer between the H5 atom of GlcA and the basic nitrogen of H85 (CV1 = d1 - d2). CV2 considers cleavage of the glycosidic bond (d4) activated by proton transfer between H85 and the glycosidic oxygen (d2 - d3), resulting in CV2 = d2 - d3 + d4. Using these two collective variables containing d2 in symmetric and antisymmetric form was the only way of simulating the multiple proton transfers involving H5 without biasing the system from evolving from reactants to products through an incorrect pathway. Preliminary tests with other collective variables did not activate both processes at the same time. To reconstruct the FEL of the β-elimination reaction, we used Gaussian potentials of 0.15 Å × 0.15 Å × 1.0 kcal/mol, adding one Gaussian every 100 MD steps, with a well-tempered parameter of 30 kcal/mol. After adding 6648 Gaussian potentials (Figure S5, 498.6 ps total simulation time), the system evolved back and forth from the Michaelis complex (MC) to the product complex (PC), crossing the transition-state region twice. After the system went back and forth from reactants to products, we considered the free-energy surface converged.

**Figure 2:**
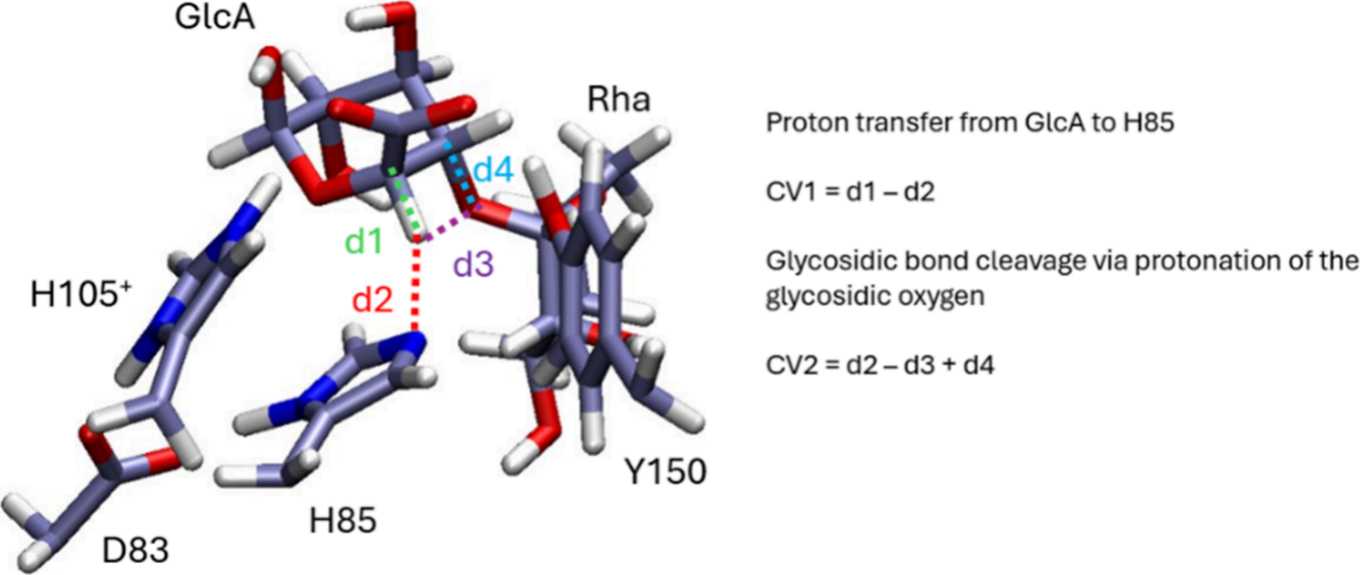
QM region of the QM/MM model and collective variable definition for the syn β-elimination metadynamics simulation.

We focused on the syn β-elimination channel because the available experimental structures place His85 and the leaving-group oxygen on the same face of the substrate and support the syn mechanism proposed from neutron crystallography.^9^ In contrast to PL38, we do not observe an obvious catalytic arrangement in FoRham1 that would support an anti abstraction. We therefore treat the syn pathway as the experimentally motivated channel to be tested here, without claiming that anti elimination is impossible in an abstract sense.

### QM/MM Umbrella Sampling Simulations

Using the same MC model as in the metadynamics simulations, we ran nine umbrella-sampling^31^ windows from CV1 = −0.75 Å to 1.25 Å in steps of 0.25 Å. Every window ran for 7.5 ps, and we used the last 5.0 ps for the weighted histogram analysis method (WHAM),^32^ which is the standard protocol used here to postprocess the information from biased umbrella-sampling simulations and construct the potential of mean force with respect to the chosen order parameter. The resulting free energy and histogram population of every window are presented in Figure S6. Also, the temporal evolution of the catalytically relevant distances for every window and the geometrical comparison between the transition state obtained with metadynamics and umbrella sampling are shown in Figure S7. In our workflow, metadynamics was used to discover the multidimensional landscape and identify the dominant reaction channel, whereas umbrella sampling was used only as a local refinement of the barrier along CV1 around that channel. The two approaches, therefore, play complementary roles rather than representing alternative, competing descriptions of the same problem.

## Results and Discussion

We simulated the reaction mechanism for the β-elimination of α-L-rhamnosyl-(1→4)-d-glucuronate by FoRham1 using QM/MM metadynamics and umbrella sampling, following the workflow summarized in the Methods section. The initial relaxed complex of FoRham1 with Rha-GlcA was obtained via classical MD simulations and QM/MM molecular dynamics simulations in aqueous solution, and the atoms of the active site included in the quantum mechanics (QM) region are shown in Figure 2. The convergence of the conformational and reaction metadynamics can be followed in Figures S4 and S5, respectively.

The conformational FEL of the GlcA moiety at subsite +1 (+1 sugar) shows a global minimum in the ^1^ *C*_4_ region, followed by another minimum in the ^5^ *S*_1_ region, 0.5 kcal mol^–1^ less stable (Figure 3). The system also explores the °*S*_2_ conformation, which is 2 kcal mol^–1^ higher in energy than the ^1^ *C*_4_. Because it is the most stable conformation and lies closest on the Cremer-Pople surface to the experimentally observed ^1^ *E* geometry of the H105F FoRham1 complex (PDB ID 7ESN),^2^ conformer ^1^ *C*_4_ was selected as the starting point for the reaction. Its plausibility is reinforced by the analogous case of GH11.^33^ Direct mutation of a residue in contact with the substrate may result in a conformational change in the hydrolyzable sugar. The previously reported H105F FoRham1 structure in complex with Rha-GlcA shows a ^1^ *E* conformation.^2^ Our study indicates that, in addition to its role in charge stabilization, H105 forms a hydrogen bond with the carboxylate group of the +1 sugar. Replacing this amino acid with a nonpolar phenylalanine depletes the enzyme–substrate H-bond interaction that may lead to the ^1^ *C*_4_ → ^1^ *E* conformational change.

**Figure 3:**
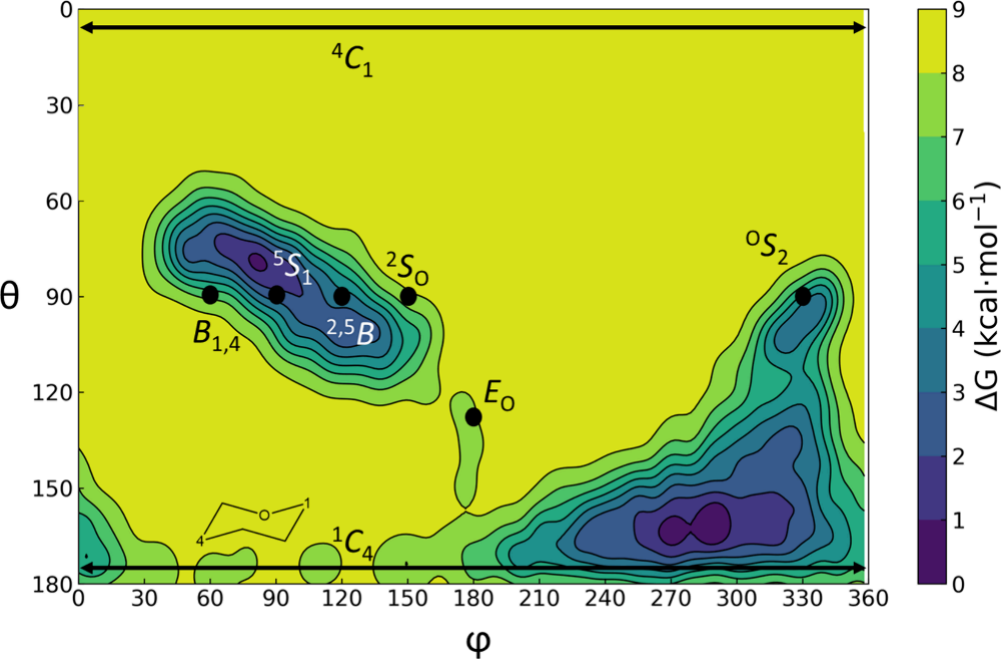
Conformational FEL of the +1 sugar (GlcA moiety) in the active site of the FoRham1 enzyme calculated via a QM/MM well-tempered metadynamics simulation.

After obtaining a representative structure of the ^1^ *C*_4_ conformer, a second QM/MM well-tempered metadynamics simulation was performed using CV1 and CV2 as collective variables (Figure 2). In Figure 4, we show the reaction FEL (left) and the projection of the minimum free energy pathway along the reaction coordinate (right). The FEL shows us the Michaelis complex (MC) minimum at around (−1.0, 1.1) Å and the product complex (PC) minimum at around (1.8, 3.4) Å. The MFEP that connects both minima passes through a saddle-point transition state (TS) at approximately (0.7, 0.3) Å. Also, we find a flat region around (1.8, 1.7) Å where an intermediate complex (IC) is located. The computed activation free energy is ΔG^‡^ = 19 kcal mol^–1^, with an overall reaction free energy of ΔG^0^ = −5 kcal mol^–1^. The IC is 1 kcal mol^–1^ more stable than the MC.

**Figure 4:**
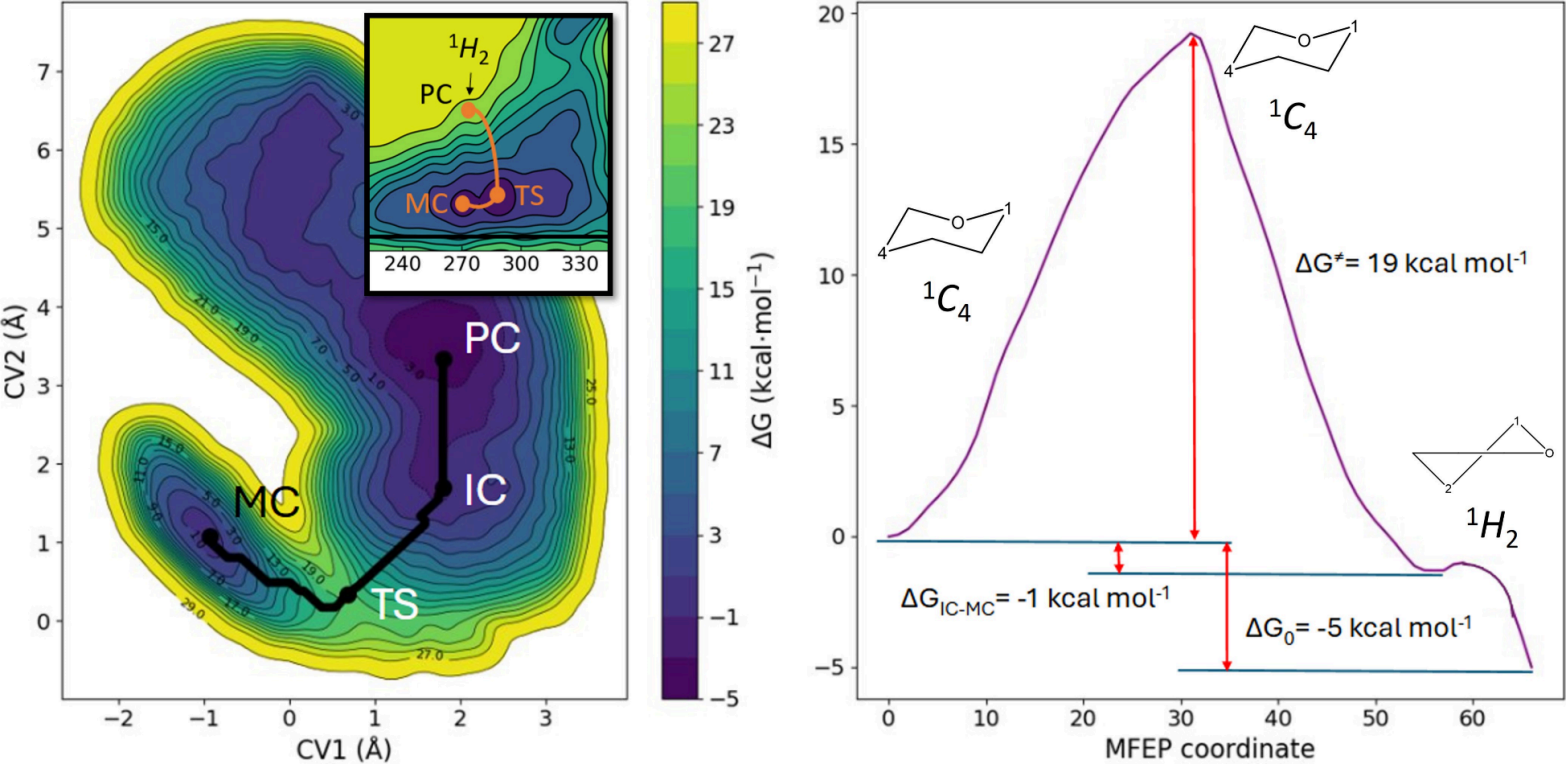
(left) Reaction FEL for the β-elimination process of Rha-GlcA in the active site of the FoRham1. The black line represents the bidimensional projection of the MFEP. Isolines are shown every 2 kcal mol^–1^. Zoom of the conformational pathway followed by the +1 sugar over the Cremer-Pople surface. (right) One-dimensional projection of the MFEP along the reaction coordinate.

The experimental activation free energy, ΔG‡, inferred from the catalytic constant is 15 kcal mol^–1^,^2^ which is 4 kcal mol^–1^ lower than our initial QM/MM estimate. Because this discrepancy is not negligible, we performed a one-dimensional umbrella sampling (US) calculation with CV1 as the collective variable (Figure S6). Along the same reaction pathway, the barrier decreases to 17.7 kcal mol^–1^, only 2.7 kcal mol^–1^ above the experimental value. The metadynamics simulation located the TS at CV1 = 0.67 Å, whereas umbrella sampling placed it within CV1 ≈ 0.60–0.80 Å. This small offset is common in metadynamics and likely reflects limited sampling of the TS region (within ∼± 1 kcal mol^–1^).

As shown in Figure 4 (upper left), we depict the conformational change of the +1 sugar along the MFEP (orange line). During the β-elimination reaction, the MC and the TS keep the same conformation (^1^ *C*_4_), and as expected, after the formation of the double bond inside the ring, the conformation of the GlcA in the PC evolves to ^1^ *H*_2_. Thus, the catalytic itinerary of the substrate is ^1^ *C*_4_ → ^1^ *C*_4_ → ^1^ *H*_2_.

Apart from the conformational itinerary, we followed the evolution of d1 = d­(H5 – C5), d2 = d­(H5 – NH85), d3 = d­(H5 – O4), d4 = d­(C4 – O4) or the glycosidic bond, d­(C5 – C6), and d­(C4 – C5) along the metadynamics of the chemical transformation and averaged them around the MFEP (CV1 ± 0.1 Å, CV2 ± 0.1 Å) (Figures 5 and 6). The result is depicted in Figure 5 (left), where the curves show an atomistic-level image of the chemical process occurring in the PL42 enzyme. Since the PL42 substrate shares structural similarity with the PL7 and PL38 ones, we have compared the evolution of d­(C5 – C6), and d­(C4 – C5) in the MC, TS, and PC structures with the ones obtained in references 12 and 13 (Figure 5 – right). We can observe similar behaviors between enzymes that will be discussed in the following lines.

**Figure 5:**
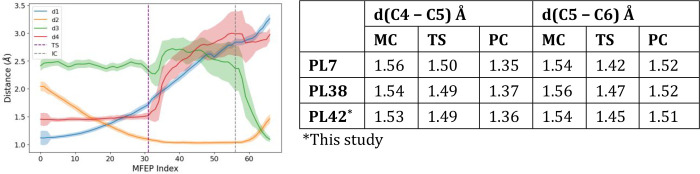
Evolution of the main catalytically relevant distances along the minimum free energy pathway for the syn β-elimination reaction of Rha-GlcA in FoRham1 (left).

**Figure 6:**
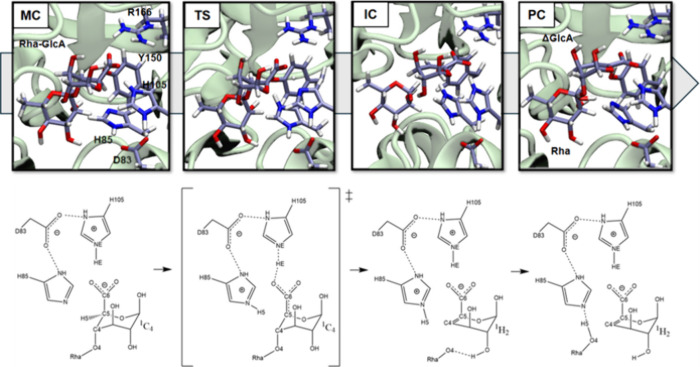
Atomistic and schematic view of the reaction mechanism followed by the Rha-GlcA substrate in the active site of the FoRham1 enzyme. Carbon, hydrogen, oxygen, and nitrogen atoms are depicted in gray, white, red, and blue, respectively.

Step by step, the mechanism shown after analyzing the MFEP evolves in the following way (Figure 6):

From MC to TS: the H5 is transferred from the C5 of the +1 GlcA moiety to the basic nitrogen of H85 (d1 vs d2). The conformation of the +1 sugar remains in ^1^C_4_ shape. As observed in Figure S8, the TS requires that the HE of H105 is shared between the amino acid and the carboxylate group (COO−) of GlcA. The C5–C6 bond acquires partial double-bond character, shortening from 1.54 to 1.45 Å.
From TS to IC: the C4 – O4 glycosidic bond (d4) is cleft in a dissociative step. The C4 – C5 bond evolves from single (1.50 Å) to double (1.37 Å). The C5–C6 bond reverts to its initial single-bond character. The H105 protonation state is restored as in MC. The glycosidic oxygen (O4) stays deprotonated and is stabilized by an intramolecular hydrogen bond to the GlcA 2-OH group. The conformation of the formed ΔGlcA moiety evolves to ^1^H_2_. The +1 sugar has a strong carbanionic character.
From IC to PC: the H5 is finally transferred from the H85 to the O4 (d2 vs d3). Access to the final ΔGlcA + Rha product.

In summary, PL42 proceeds via a concerted yet asynchronous syn β-elimination, passing through a carbanion-like transition state in which charge is delocalized over C4–C5–C6. A hydrogen-bonding, proton-rich environment around the GlcA carboxylate—shaped primarily by H105 and R166 ^9^—helps stabilize this electronegative intermediate/TS and thereby lowers the activation barrier for FoRham1.

Overall, our simulations support syn β-elimination as a viable pathway for FoRham1-catalyzed cleavage of Rha-GlcA. QM/MM metadynamics identifies the ^1^ *C*_4_ reactive conformation as the catalytically competent starting state, and this state lies close on the Cremer-Pople surface to the experimentally observed ^1^ *E* geometry of the H105F mutant,^2^ without implying identity between the two conformers. Our simulation of the syn β-elimination shows an activation and thermodynamic barrier of 19 kcal mol^–1^ and −5 kcal mol^–1^, respectively. Although the reverse reaction has not been tested experimentally, we do not overinterpret the absolute thermodynamic value beyond noting that the product state is slightly stabilized relative to the Michaelis complex. The 4 kcal mol^–1^ difference from the experimental barrier (15 kcal mol^–1^) likely reflects both limited TS sampling in metadynamics (∼1.3 kcal mol^–1^ relative to umbrella sampling) and intrinsic QM/MM uncertainties, including the choice of DFT functional and basis set and the definition of the QM region. Our calculations found that the PL42 enzyme follows a ^1^ *C*_4_ → ^1^ *C*_4_ → ^1^ *H*_2_ conformational pathway. As proposed by Yano et al.,^9^ H85 serves as the catalytic base, abstracting the H5 proton and thereby promoting glycosidic bond cleavage. Transition-state stabilization relies on the H85–H105-D83 triad, with H105 forming a shared-proton interaction with the substrate carboxylate. Furthermore, R166 is key to counterpoise the charge of the carboxylate group and keep the substrate in the reactive conformation (the functional groups of a ^1^ *C*_4_ conformer of GlcA are all in axial orientation) via two H-bond interactions.

But how does the PL42 enzyme fit into the current overall view of polysaccharide lyases? Until now, PL7 is shown to follow a syn β-elimination mechanism on β-D-mannuronate activated by a deprotonated tyrosine following a ^4^ *C*_1_ → ^2^ *S*_O_ → ^2^ *H*_1_ pathway,^12^ and PL38 can act on α-L-guluronate via anti β-elimination (with a histidine acting as base and a tyrosine acting as acid) or on β-D-mannuronate via syn β-elimination (with a tyrosine acting as base) and both mechanisms follow a ^2^ *S*_O_ → *B*_3,O_ → *B*_3,O_ path.^13^ In this work, the PL42 enzyme cleaves α-d-glucuronate via syn β-elimination driven by a catalytic histidine base and proceeds along a ^1^ *C*_4_ → ^1^ *C*_4_ → ^1^ *H*_2_ conformational itinerary.

In parallel with glycoside hydrolases, where the oxocarbenium-like transition state keeps four atoms in the same plane (C5, O5, C1, and C2),^34^ the product of a PL contains a double bond, which restricts, typically, four atoms in the same plane (in the case of ΔGlcA, O5, C5, C4, and C3). As Alonso-Gil demonstrated in 2020, six-membered rings with a restricted dihedral angle can explore a linear and continuous space on the Cremer and Pople^35^ Mercator surface.^36^ For the PL product, the potential canonical conformations are ^3,O^ *B*, *E*_1_, ^2^ *H*_1_, ^2^ *E*, *B*_3,O_, ^1^ *E*, ^1^ *H*_2_, and *E*_2_ (Figure 7). Connecting these eight points on the Cremer-Pople surface yields a linear, continuous relation, θ = 90 + (90–51) cos­(φ-270), which defines the end point common to all PL mechanisms (Figure 7).

**Figure 7:**
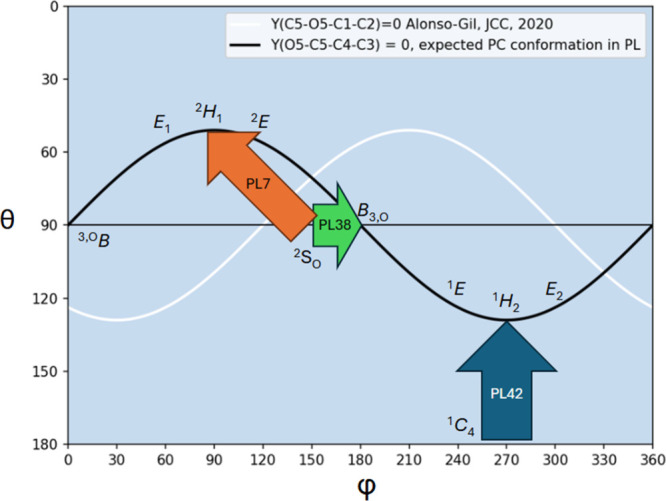
Comparison of the conformational pathways observed for the three polysaccharide lyases modeled using QM/MM enhanced sampling methods. In comparison with glycoside hydrolases (GHs), where a reaction mechanism can be understood by the conformation of the transition state (the sugar in subsite −1 has a configuration where the C5, O5, C1, and C2 ring atoms are coplanar), polysaccharide lyases can be classified according to the conformation of the product ring (in this case, O5, C5, C4, and C3 are coplanar).

## Conclusions

Our combined QM/MM well-tempered metadynamics and umbrella sampling simulations support a concerted, highly asynchronous syn β-elimination mechanism for PL42 (FoRham1) that is fully compatible with the available kinetic data. The reaction proceeds along a ^1^ *C*_4_ → ^1^ *C*_4_ → ^1^ *H*_2_ conformational itinerary of the GlcA unit at subsite +1, with H85 acting as the catalytic base, assisted by the D83–H105 pair, and R166 providing electrostatic stabilization of the uronate carboxylate. The free energy barrier obtained from the converged 1D-US profile agrees well with the experimental activation energy, lending confidence to the proposed reaction coordinate and TS structure.

A direct comparison with recent QM/MM studies of PL7 and BoPL38 highlights both common principles and family specific solutions to β-elimination catalysis. All three enzymes stabilize a carbanion-like transition state at subsite +1, characterized by concomitant shortening of the C4 – C5 and C5 – C6 bonds. However, while PL7 and BoPL38 rely on a preactivated ^2^ *S*_O_ conformation of the +1 sugar and a tyrosine as general base (alone or in combination with a histidine residue), PL42 exploits an intrinsically “preactivated” ^1^ *C*_4_ conformation of GlcA and a histidine-based catalytic triad. These differences, together with the distinct product conformations (^2^ *H*_1_ for PL7, *B*_3,O_ for BoPL38, and ^1^ *H*_2_ for PL42), can be rationalized in terms of a single linear pathway on the Cremer–Pople surface that connects all observed PL product conformations.

This “PL path” provides a simple conformational framework to classify β-elimination mechanisms in polysaccharide lyases, complementary to the well-established transition-state-based scheme used for GHs. By placing PL42 on this map alongside PL7 and BoPL38, our work extends the emerging unified view of PL catalysis, suggesting clear starting points for engineering PL42-family enzymes with altered specificity or activity, as well as for designing mechanism-based inhibitors that target the carbanion-like TS.

## Supplementary Material



## Data Availability

All input files, substrate antechamber parameters, and analysis scripts used in this work are available at GitHub https://github.com/drsalonsogil/PL42_QMMM_MD_simulations.git.

## Notes

Supporting Information contains Figures S1–S8 and the extra distance analysis referred to in the main text (PDF)

## Abbreviations

*B*: Boat
*C*: Chair
CV1: Collective variable 1
CV2: Collective variable 2
DFT: Density functional theory
*E*: Envelope
FEL: Free energy landscape
GA: Gum arabic
GH11: Glycoside hydrolase family 11
*H*: Half-chair
IC: Intermediate complex
MC: Michaelis complex
MD: Molecular dynamics
MFEP: Minimum free energy pathway
PC: Product complex
PDB: Protein Data Bank
PL: Polysaccharide lyase
PL38: Polysaccharide lyase family 38
PL42: Polysaccharide lyase family 42
PL7: Polysaccharide lyase family 7
QM/MM: Quantum mechanics/molecular mechanics
Rha-GlcA: α-L-rhamnosyl-(1→4)-d-glucuronate
*S*: Skew-boat
TS: Transition state
US: Umbrella sampling
