# Supplementary material for: Untangling the Reaction Mechanism of the Polysaccharide Lyase PL42 Using QM/MM Metadynamics Simulations
Source: J Chem Inf Model. 2026 Apr 17;66(9):5463–70. doi: 10.1021/acs.jcim.6c00945 (PMC13169297; doi:10.1021/acs.jcim.6c00945)
Supplement: Supplementary file 1 [file ci6c00945_si_001.pdf]

Supporting Information

Untangling the reaction mechanism of the polysaccharide lyase PL42 using QM/MM metadynamics simulations

Santiago Alonso-Gil,[1,2*] Tatsuya Kondo,[3] Shinya Fushinobu,[4,5] and Pedro A. Sánchez-Murcia,[1,6*]

[1] Laboratory of Computer-Aided Molecular Design, Division of Medicinal Chemistry, Otto-Loewi Research Center, Medical University of Graz, Neue Stiftintalstr. 6/III, A-8010 Graz, Austria
[2] Institut Laue-Langevin, Grenoble, France
[3] Department of Applied Biological Chemistry, Graduate School of Agriculture, Osaka Metropolitan University, Sakai, Osaka 599-8531, Japan
[4] Department of Biotechnology, The University of Tokyo, Bunkyo-ku, Tokyo 113-8657, Japan
[5] Collaborative Research Institute for Innovative Microbiology, The University of Tokyo, Bunkyo-ku, Tokyo 113-8657, Japan
[6] BioTechMed-Graz, Mozartgasse 12/II, A-8010 Graz, Austria
Corresponding authors: S. Alonso-Gil: santiago.alonso-gil@medunigraz.at, P. A. Sánchez-Murcia: pedro.murcia@medunigraz.at

Contents: Figures S1–S8 and extra distance analysis.

Supporting Figures

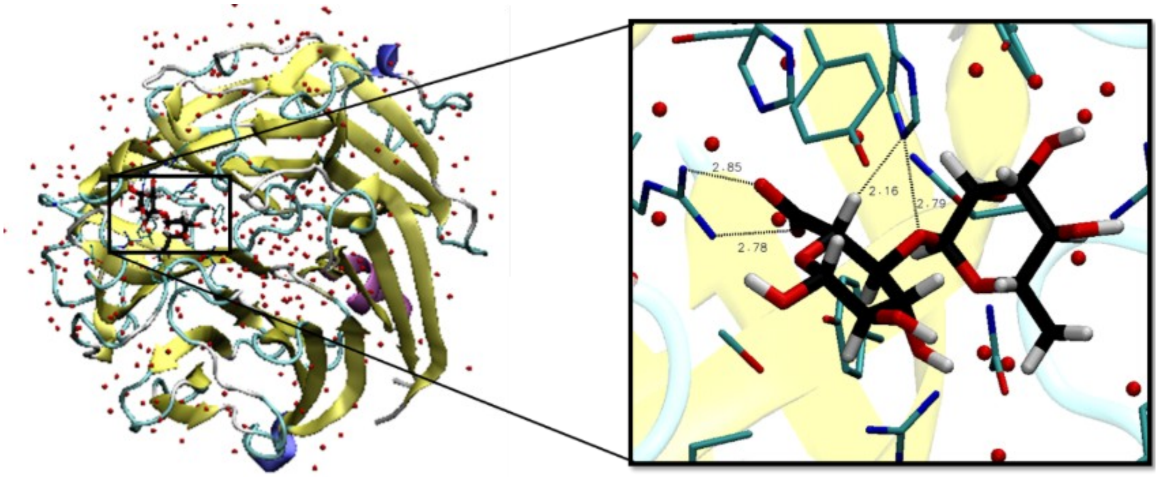

Figure S1. Three-dimensional representation of the reconstructed (left) PL42-Rha-GlcA model and (right) its active site.

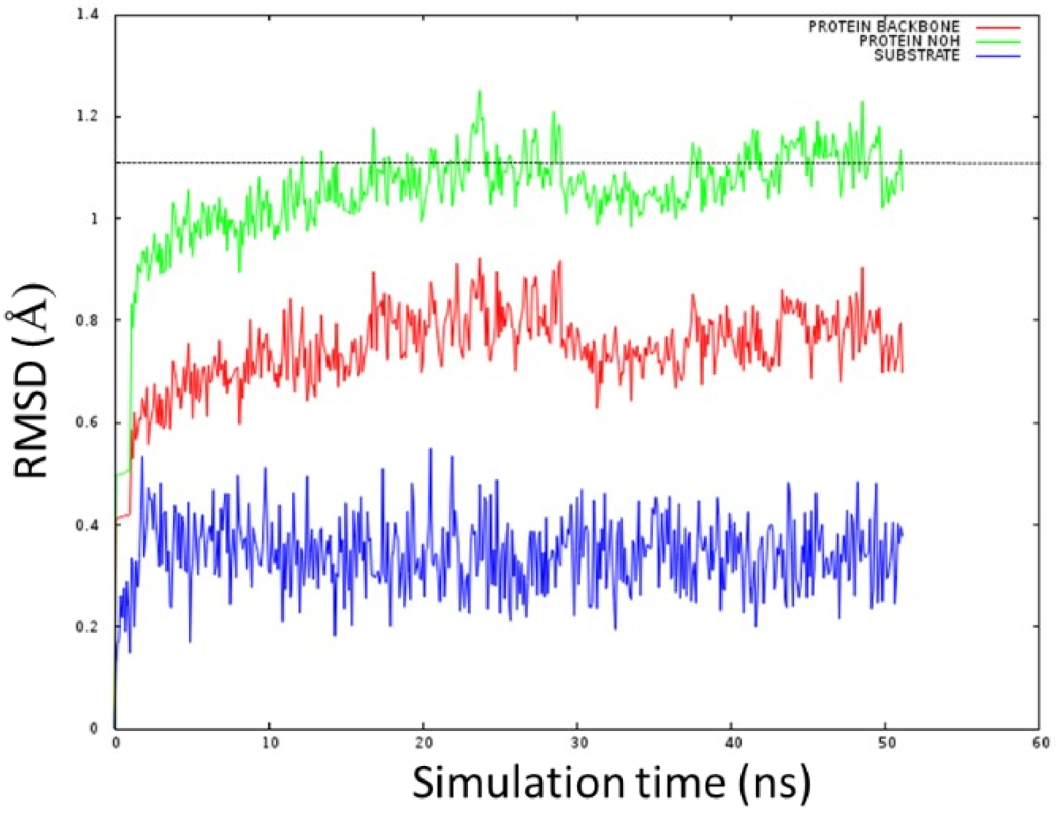

Figure S2. RMSD evolution along the 50 ns equilibration MD of the PL42-Rha-GlcA model.

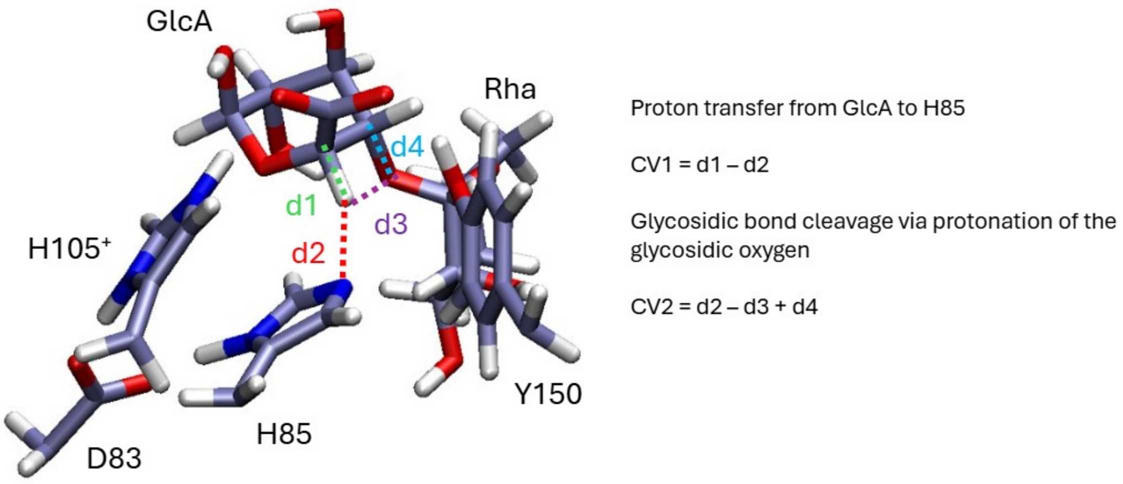

Figure S3. QM region of the QM/MM model and collective variable definition for the syn β-elimination metadynamics simulation.

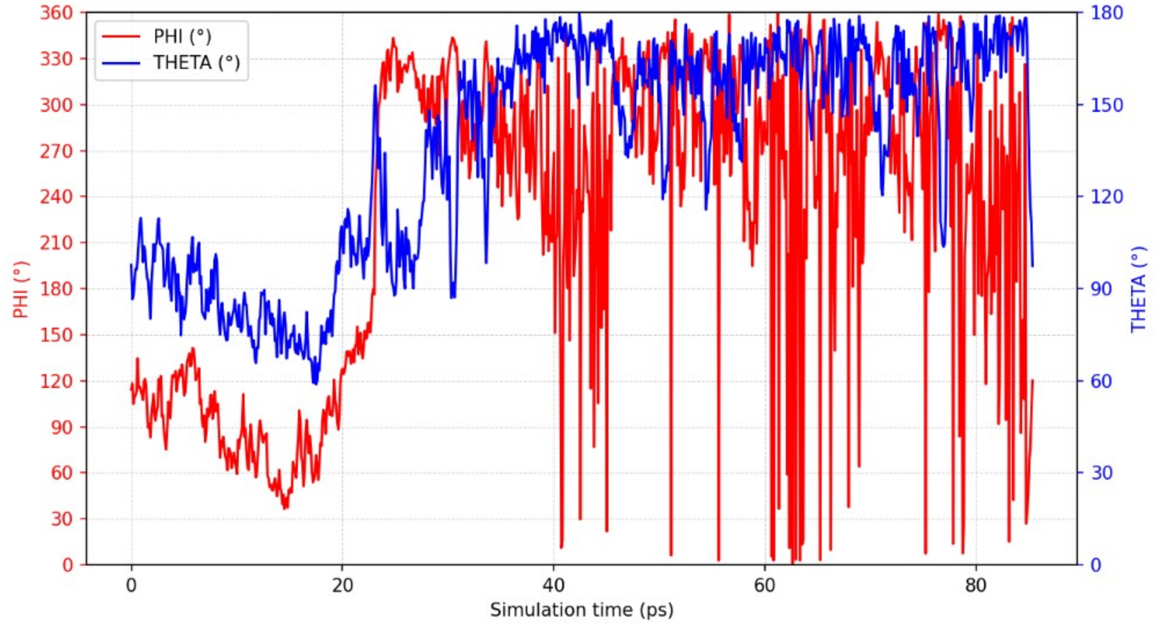

Figure S4. Time evolution of the puckering coordinates of the +1 sugar in the FoRham1 complex along the QM/MM well-tempered metadynamics simulation.

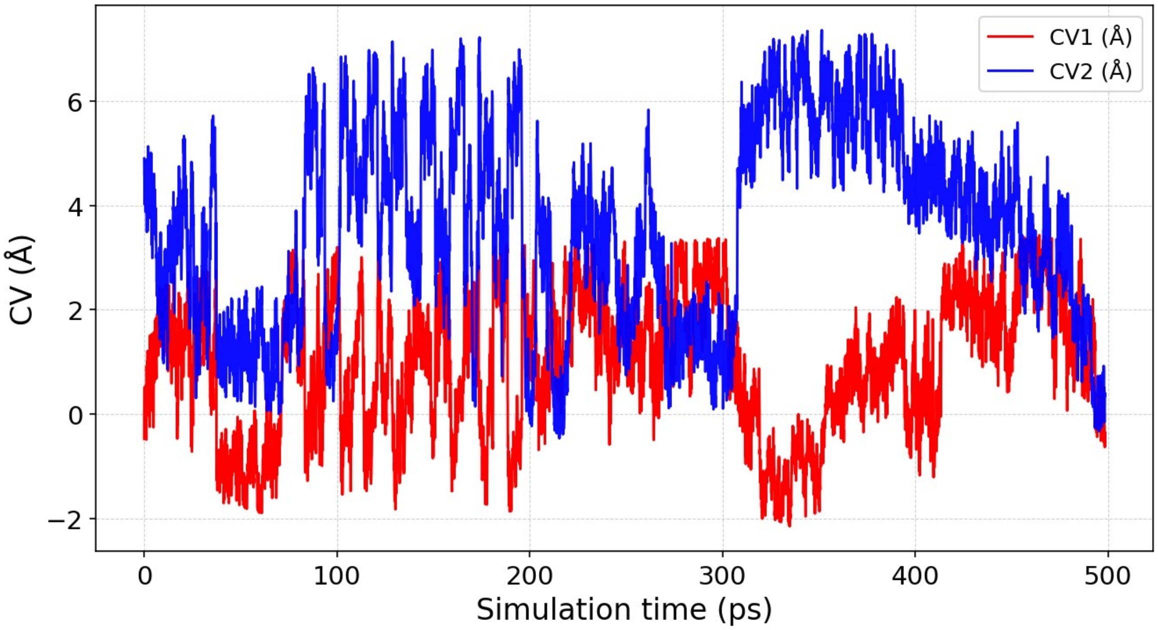

Figure S5. Time evolution of the CV1 (red) and CV2 (blue) collective variables along the QM/MM well-tempered metadynamics simulation of the syn β-elimination reaction in FoRham1.

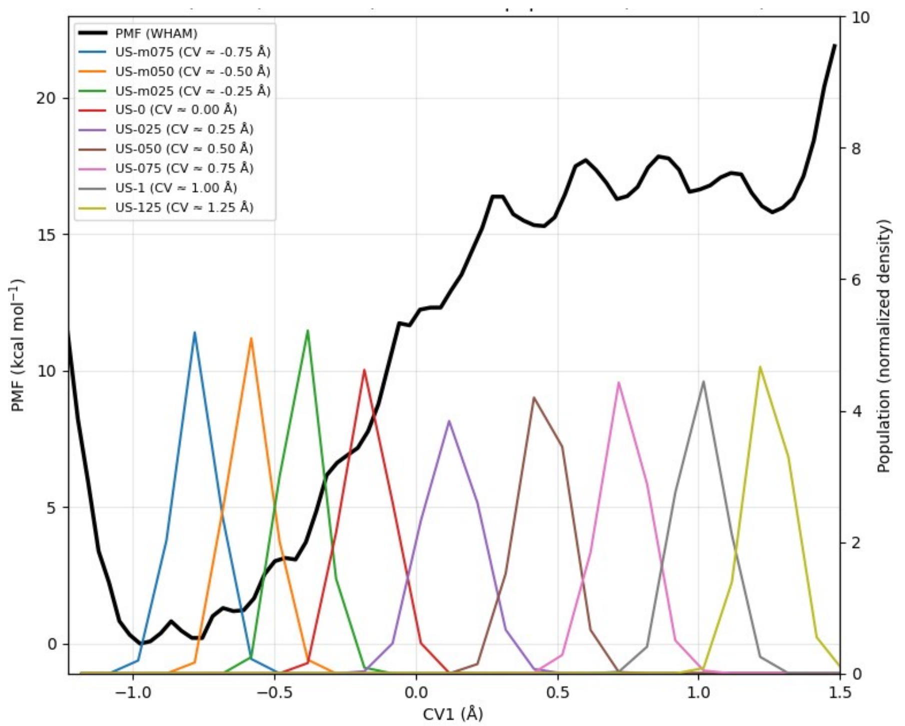

Figure S6. Monodimensional representation of (black line) the potential of mean force (PMF, kcal mol-1) associated with the H5 transfer from GlcA to H85 in FoRham1. The normalized population of every umbrella-sampling window is shown in color.

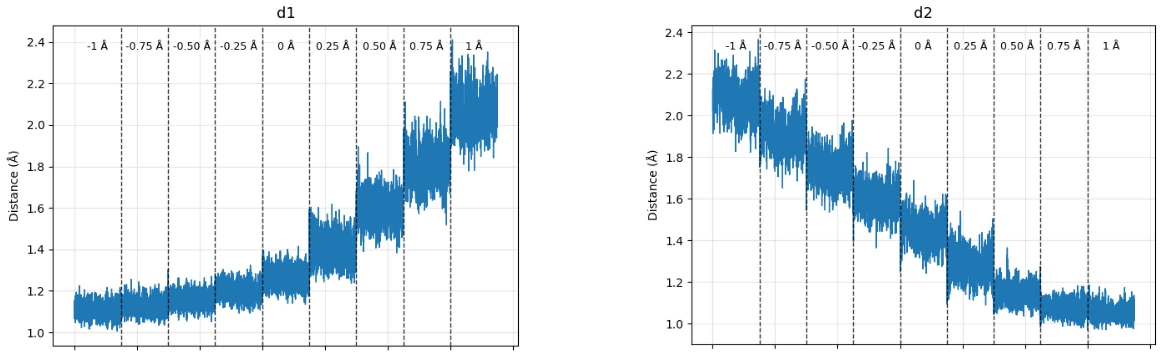

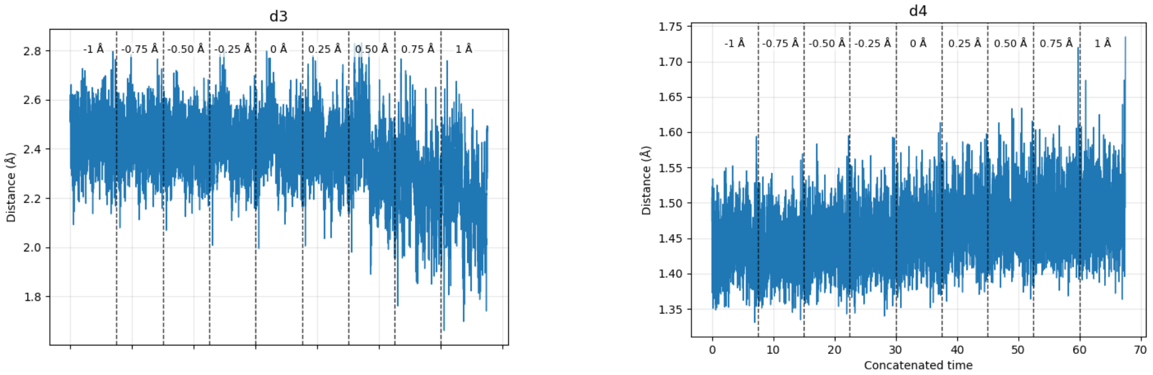

	**<d1>**	**<d2>**	**<d3>**	**<d4>**
**TS – metadynamics**	**1.7 ± 0.1**	**1.1 ± 0.1**	**2.4 ± 0.2**	**1.5 ± 0.2**
**TS - US**	**1.6-2.0**	**1.1 ± 0.1**	**2.3 ± 0.2**	**1.5 ± 0.1**

Figure S7. (Up) Temporal evolution of the catalytically relevant distances d1-d5 in Å along the 9 umbrella sampling windows and (down) geometrical comparison between the transition states found using metadynamics and umbrella sampling simulations.

Extra distance analysis

Our statistical study contains information on some of the most catalytically relevant distances along the metadynamics simulation. However, two relevant distances were not monitored using PLUMED: the distances related to a potential proton transfer between the Nε atom of H105 and the carboxylate (COO-) group of the +1 sugar. We therefore ran three unrestrained QM/MM MD simulations of 7.5 ps using representative snapshots of the MC, IC, and PC, and combined them with one of the trajectories obtained during the umbrella-sampling study (CV1 = 0.5 Å) to monitor how the distance between H105-HE changes with respect to the carboxylate group and to H105 itself.

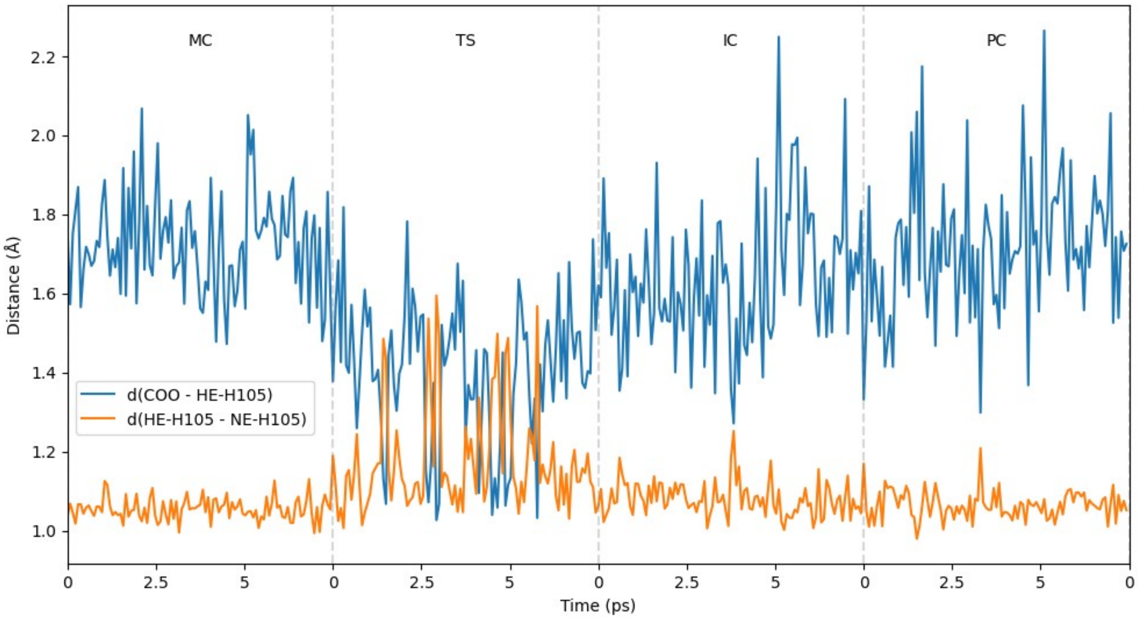

Figure S8. Temporal evolution of the distances between H105-HE with respect to the COO- group of the +1 sugar and the H105 residue.
